# How severe acute respiratory syndrome coronavirus-2 aerosol propagates through the age-specific upper airways

**DOI:** 10.1063/5.0061627

**Published:** 2021-08-19

**Authors:** Mohammad S. Islam, Puchanee Larpruenrudee, Suvash C. Saha, Oveis Pourmehran, Akshoy Ranjan Paul, Tevfik Gemci, Richard Collins, Gunther Paul, Yuantong Gu

**Affiliations:** 1 School of Mechanical and Mechatronic Engineering, University of Technology Sydney (UTS), 15 Broadway, Ultimo, New South Wales 2007, Australia; 2 School of Mechanical Engineering, The University of Adelaide, Adelaide, South Australia 5005, Australia and Department of Surgery—Otolaryngology Head and Neck Surgery, The University of Adelaide, Adelaide, South Australia 5005, Australia; 3 Department of Applied Mechanics, Motilal Nehru National Institute of Technology Allahabad, Prayagraj 211004, Uttar Pradesh, India; 4 Braun Medical, Irvine, California 92614, USA; 5 Biomechanics International, Cranberry Township, Pennsylvania 16066, USA; 6 James Cook University, Australian Institute of Tropical Health and Medicine, Townsville, Queensland 4810, Australia; 7 School of Mechanical, Medical and Process Engineering, Faculty of Engineering, Queensland University of Technology, Brisbane 4000, Australia

## Abstract

The recent outbreak of the COVID-19 causes significant respirational health problems, including high mortality rates worldwide. The deadly corona virus-containing aerosol enters the atmospheric air through sneezing, exhalation, or talking, assembling with the particulate matter, and subsequently transferring to the respiratory system. This recent outbreak illustrates that the severe acute respiratory syndrome (SARS) coronavirus-2 is deadlier for aged people than for other age groups. It is evident that the airway diameter reduces with age, and an accurate understanding of SARS aerosol transport through different elderly people's airways could potentially help the overall respiratory health assessment, which is currently lacking in the literature. This first-ever study investigates SARS COVID-2 aerosol transport in age-specific airway systems. A highly asymmetric age-specific airway model and fluent solver (ANSYS 19.2) are used for the investigation. The computational fluid dynamics measurement predicts higher SARS COVID-2 aerosol concentration in the airway wall for older adults than for younger people. The numerical study reports that the smaller SARS coronavirus-2 aerosol deposition rate in the right lung is higher than that in the left lung, and the opposite scenario occurs for the larger SARS coronavirus-2 aerosol rate. The numerical results show a fluctuating trend of pressure at different generations of the age-specific model. The findings of this study would improve the knowledge of SARS coronavirus-2 aerosol transportation to the upper airways which would thus ameliorate the targeted aerosol drug delivery system.

## I. INTRODUCTION

The outbreak of severe acute respiratory syndrome (SARS) coronavirus-2, commonly known as COVID-19 has, in late 2019, posed different challenges to different sociodemographic groups. The disease has hit the elder population harder than any other age groups because of the latter's pre-existing comorbidities; viz., hypertension, diabetes, cardiovascular diseases, chronic pulmonary and even renal diseases, etc. (Al-Zahrani, 2021; Smorenberg *et al.*, 2021). Aging changes respiratory physiology, pathology and their working, more often irreversibly, posing a serious concern especially during pulmonary infection. Subsequently, it affects the sensitivity and survivability of the aged patients, eventually make them more vulnerable to COVID-19 (Lowery, 2013; Miller and Linge, 2017; Liu *et al.*, 2020a). More than 95% of the COVID-19 deaths considered in this study included populations over 60 years of age, while more than 50% of deaths included people over 80 years of age as revealed in WHO data (Sandoiu, 2021). Due to many degenerative processes taking place in the body, vital capacity (i.e., the total amount of air exhaled after maximal inhalation) of the lungs of the aged people is decreased and, hence, it requires excess work to maintain normal breathing (Stocks and Quanjer, 1995).

Researchers investigated the relation of aging to the severity of COVID-19 for patients located in Australia (Holt *et al.*, 2020), Israel (Clarfield and Jotkowitz, 2020), China (Guo *et al.*, 2020), India (Singh *et al.*, 2020) and in many other countries (Ayoub *et al.,* 2020; Davies *et al.*, 2020). It is found that the fatality rate of COVID-19 patients in countries such as Italy, Spain, and China were severely affected by SARS coronavirus-2 pandemic in its first outbreak and increased exponentially with the age of the patients (Santesmasses *et al.*, 2020). Possible remedies were also suggested by some researchers (Koff and Williams, 2020; Perrotta *et al.*, 2020; Mueller *et al.*, 2020) for elderly patients, which include those in long-stay residential care homes and hospitals. Among all, the SARS viruses and their SARS coronavirus-2 variants can reach the lower airways, causing severe damage to pulmonary tissues, thus turning them from spongy to stiffer (fibrosis and scarring) forms, subsequently resulting in high fatality from pneumonia (Zhu *et al.,* 2020). A recent study by Edwards *et al.* (2021) suggests that the formation of droplets from the mucus lining of human airways and aerosol exhalation increases with acute COVID-19 infection and patients' body mass index (BMI) multiplied with increased age. The susceptibility to disintegrated mucus layers in the airway lining is much higher in aged populations, making aged patients superspreaders. Aging is an irreversible and degenerative phenomenon. Various physiological and immunological factors that are responsible for aging of the human respiratory system are discussed by researchers (Rossi *et al.*, 1996; Pride, 2005; Sharma and Goodwin, 2006). Aging of human airways is reflected by changes in material (elasticity) and anatomical (lumen diameter and wall thickness) properties because of morphological and tissue variations (Lai-Fook and Hyatt, 2000). The diameter of smaller airways (bronchioles < 2 mm) becomes narrow with age for people aged over 40 years and a 10% reduction in bronchioles is seen for people aged between 50 and 80 years (Niewoehner and Kleinerman, 1974). The lungs are never fully empty, and the amount of air volume left after complete expiration is termed residual volume (RV). With age, the residual volume increases, mainly due to the reduction in airway diameters (Leblanc, 1970). The forced vital volume (FVC) for expiration during a spirometry test is found to be reduced by 50% due to intrinsic reduction in the mean diameter of the membranous bronchioles (Knudson, 1991). The airway walls are thickened due to the increase in collagen and membranes (Montaudon *et al.*, 2007) in the lungs of older people suffering from severe asthma (Bai *et al.*, 2000). Hence, elderly patients with a history of respiratory illness are more vulnerable to COVID-19 infection. Changes due to aging (compliance of lung and mechanical properties of airways) (Kim *et al.,* 2017) increase the chances of lung injury during mechanically ventilated procedures.

Respiratory routes are preferred for administering drugs for both pulmonary as well systematic diseases (Jaafar-Maalej *et al.*, 2009). Adapting the morphological properties of lungs into the respiratory tract models, selection of ventilation parameters (breathing patterns, etc.), aerosol characteristics (size, shape, and material properties), growth of obstacles (lesions/tumors) in the airways, fluid–wall interactions, and in the *in vitro* studies are essential in predicting the performance of aerosol transport (Thompson, 1998; Labiris and Dolovich, 2003; Srivastav *et al.*, 2019a; Shukla *et al.*, 2020; Singh *et al.*, 2021). Various researchers (Srivastav *et al.*, 2013; Srivastav *et al.*, 2014; Islam *et al.*, 2017a; Islam *et al.,* 2018) investigated the particle and aerosol transport to human airways, providing insight into the absorption and deposition mechanisms of inhaled drugs with a view of designing better drugs for targeted drug delivery through pulmonary routes. Many researchers (Kim *et al.*, 2015; Das *et al.*, 2018; Ostrovski *et al.*, 2019; Tiwari *et al.*, 2021a; Srivastav *et al.*, 2021; Tiwari *et al.*, 2021b) focused on targeted delivery into the lungs using experimental as well as computational fluid dynamics (CFD) methods to study the aerosol transport dynamics leading to the development of better inhalation devices. Recently, Islam *et al.* (2020) reviewed the efficacy of various numerical models available to estimate deposition of aerosols in the human airways.

Amid the outbreak of COVID-19, the focus has now shifted to the investigation of the transmission of SARS coronavirus-2 viruses in the form of aerosols and droplets in public places and to the respiratory tract. A plethora of research has been conducted in recent times to study the transmission behavior of SARS coronavirus-2 in various public places, such as elevators (Dbouk and Drikakis, 2021), escalators (Li *et al.*, 2021a), dental clinics (Li *et al.*, 2021b), hospital isolation rooms (Bhattacharyya *et al.*, 2020), vehicle parking areas (Nazari *et al.*, 2021), buses (Zhang *et al.*, 2021), passenger aircraft (Talaat *et al.*, 2021), classrooms (Foster and Kinzel, 2021), restaurants and cafeterias (Liu *et al.*, 2021, Wu *et al.*, 2021), conference rooms (Mirikar *et al.*, 2021), classrooms (He *et al.*, 2021), public restrooms (Schreck *et al.*, 2021), in a city (Zheng *et al.*, 2021), and even during a face-to-face scenario with an utterance (Ishii *et al.*, 2021). On the other hand, Jarvis (2020) found that the SARS coronavirus-2 borne aerosol particles can combine with particulate matter (PM) present in the atmosphere, possibly leading to even higher infection rates. Habitual cigarette smokers also face higher risks of toxic particle deposition in the distal regions of the respiratory tract (Paul *et al.*, 2021). Tang *et al.* (2020) and Mutuku *et al.* (2020) confirmed that the plausibility of aerosol transmission for SARS coronavirus-2 viruses is very high. Hence, suitable strategies are discussed by researchers to contain or minimize airborne transmission of atmospheric viral loads through ventilation (Jarvis, 2020), such as in hospitals and isolation rooms (Bhattacharyya *et al.,* 2020). Human nasal passages can be of 5–10 *μ*m in the lumen. The airways are, however, reduced to 2.5–5 *μ*m in the trachea (TR) and to 0.1–2.5 *μ*m in further downstream locations. Guzman (2021) revealed that the size of the particles laden with SARS coronavirus-2 RNA can be as small as 0.25–1 *μ*m, which can often be readily inhaled into the respiratory tract and could easily be transmitted airborne by diffusion. On the other hand, larger particles containing viral loads (2.5–10 *μ*m) have a chance to deposit in the nasal, oropharyngeal, laryngeal, and tracheal regions of the respiratory system due to gravitational settling and cause infection. Mallik *et al.* (2020) conducted experiments to study the aerosol transport in bronchioles and found that the aerosol deposition increases with the bronchiole diameter. It was also pointed out that a high breath-hold time can increase the possibility of viral infection, especially in a crowded place as it promotes further aerosol deposition in the alveoli.

It is evident that the airway diameter reduces with age and a precise understanding of the SARS coronavirus-2 aerosol transport through pulmonary airways of different elderly people's airways could potentially help in improving the overall respiratory health assessment that is lacking in the literature. The present study aims to numerically investigate the SARS coronavirus-2 aerosol transportation to an age-specific airway system for the first time.

## II. NUMERICAL METHODS

This study considered steady laminar flow and analyzed the SARS coronavirus-2 aerosol transportation to the upper airways of an age-specific lung. ANSYS-FLUENT (v.19.2) solver and Lagrangian approach are employed to solve the fluid flow and particle transport equations. The study solved the steady mass and momentum equations,

$$\nabla .(\rho \vec{v})=0,$$ (1)

$$\nabla .(\rho \vec{v}\vec{v})=-\nabla p+\nabla .(\mu (\nabla \vec{v}+\nabla {\vec{v}}^{T}))+\rho \vec{g},$$ (2)

where $\rho \vec{g}$ and *p* denote the gravitational body force and static pressure, respectively. The molecular viscosity is defined as *μ*.

The numerical study solved the internal energy equation for Brownian motion of the nanosized SARS coronavirus-2 aerosol,

$$\nabla \cdot \left(\rho \vec{v}e\right)\;=-\nabla \cdot \vec{J},$$ (3)

where *e* is the specific internal energy and $\vec{J}$ is the heat flux.

The inhalation condition at the mouth–throat (MT) region is highly complex, and there is no established velocity profile for inhalation. Studies to date mostly used uniform inlet conditions for one-way inhalation (Islam *et al.*, 2021a; Kuprat, 2021; Zhao *et al.*, 2021) and parabolic (Bass *et al.*, 2021; Chen *et al.*, 2021; Fan *et al.*, 2021; Hayati *et al.*, 2021). During inhalation, the uniform flow field could become highly turbulent at the mouth–throat area at a flow rate of >30 lpm (Islam *et al.*, 2018; Islam *et al.*, 2019; Kleinstreuer and Zhang, 2003; Zhang *et al.*, 2005), and the flow profile becomes parabolic at the trachea and upper airways (Gemci *et al.*, 2008; Islam *et al.*, 2017). This study used uniform inlet conditions at the mouth–throat inlet. The overall SARS coronavirus-2 aerosol transportation to the mouth and upper airways is analyzed for the flow rates of 7.5 and 15 lpm. The outlet condition at the truncated outlets is pressure-based, and zero pressure is assigned at the end of the branches. This study did not consider all the bifurcations; only the first five bifurcations were considered in the analysis. Therefore, the open outlet condition used at the outlet is adopted from the published literature (Cui *et al.*, 2021; Islam *et al.*, 2021a).

The scatter in the particulate size of SARS coronavirus-2 is tiny, and in the isolated condition, the size is about 120 nm (https://www.pptaglobal.org/media-and-information/ppta-statements/1055–2019-novel-coronavirus-2019-ncov-and-plasma-protein-therapies). However, a recent study shows that the virus size could vary, and it could be up to 1000 nm (Liu *et al.*, 2020b). The isolated SARS coronavirus-2 viruses could aggregate or transport as droplets during exhalation, coughing, or sneezing. Therefore, the overall diameters of the virus-laden aerosol will increase, which is evident in the literature (Chirizzi *et al.*, 2021). This study used three different sized (120 nm, 500 nm, and 1 *μ*m) SARS coronavirus-2 aerosols and analyzed the deposition at the upper bronchioles for aged lungs.

Therefore, the particle transport equations include Brownian motion for the nanosize SARS coronavirus-2 aerosol (Inthavong *et al.*, 2009; Islam *et al.*, 2021b),

$$\begin{array}{c} \frac{du_{i}^{p}}{dt}=F_{D}+F_{\mathrm{Brownian}}+F_{\mathrm{Lift}}+\frac{\rho_{p}-\rho_{g}}{\rho_{p}}g_{i},\\ \;F_{D}=\frac{1}{C_{c}}C_{D}A_{p}\frac{\rho_{g}\left|u_{i}^{g}-u_{i}^{p}\right|(u_{i}^{g}-u_{i}^{p})}{2m_{p}}=\frac{18\mu_{g}}{\rho_{p}d_{p}^{2}C_{c}}(u_{i}^{g}-u_{i}^{p}),\\ \;C_{c}=1+\frac{2\lambda }{d_{p}}(1.257+0.4e^{\frac{-0.11d_{p}}{2\lambda }}), \end{array}$$ (4)

where $F_{D}$ is the force due to drag, drag coefficient is $C_{D}$, $A_{p}$ is the aerosol area, and $C_{c}$ is the Cunningham factor. The $C_{c}$ value for 120 nm, 500 nm, and 1 *μ*m aerosols are 2.52, 1.39, and 1.19, respectively. λ is gas molecules' mean free path. $\rho_{p}$ is the aerosol density, and $\rho_{g}$ is the air density, respectively. $g_{i}$ is the gravitational term. $\mu_{g}$ denotes the gas viscosity and $d_{p}$ is the aerosol diameter. For the low Reynolds number of the particle (Rep<0.5), the drag coefficient *C_D_* is defined as (Haider and Levenspiel, 1989)

$$C_{D}=\frac{24}{{Re}_{p}},\;{Re}_{p}<0.5,$$ (5)

where the particle Reynolds number

$${Re}_{p}=\rho_{g}\frac{d_{p}\left|u_{r}\right|}{\mu_{g}},$$ (6)

where *u_r_* is the relative velocity. The Brownian force amplitude is

$$F_{\mathrm{Brownian}}=\zeta \sqrt{\frac{\pi S_{0}}{\Delta t}},$$ (7)

where *ζ* is the unit Gaussian random number variance and *Δt* is the time step integration of the aerosol. The spectral intensity (*S_0_*) is defined as

$$S_{o}=\frac{216\mu k_{B}T}{\pi^{2}\rho_{p}d_{p}^{5}{\left(\frac{\rho_{p}}{\rho_{g}}\right)}^{2}C_{c}}.$$ (8)

Here, *T* is the absolute temperature of the gas, *k_B_* is defined as the Boltzmann constant, and *ρ_g_* is the air density.

The numerical study used the SIMPLE scheme for pressure and velocity coupling. The Second-order discretization technique is employed for energy and momentum equations (Kumar *et al.*, 2019). SARS coronavirus-2 aerosol particles are considered spherical (https://www.nih.gov/news-events/nih-research-matters/novel-coronavirus-structure-reveals-targets-vaccines-treatments). The available literature (Goldsmith *et al.*, 2020) reports that SARS coronavirus-2 aerosol particles are spherical and consist of dark dots on the spherical shape. Therefore, spherical SARS coronavirus-2 aerosols are used for the analysis, and the density used is 1.0 g/cm^3^ (Islam *et al.*, 2021b). The SARS coronavirus-2 aerosols are injected from the mouth–throat inlet, and all aerosols are inserted only once. The SARS coronavirus-2 aerosol distribution is uniform as the velocity, and monodisperse aerosols are injected. The number of particles convergence was also tested for various sets of particles, and a total of 13 460 particles were injected from the inlet face for the final analysis. The interaction between air and SARS coronavirus-2 aerosols is considered. The residual convergence criterion for the continuity equation is 0.0001 (10^−4^), and for the energy equation, it is 0.000 001 (10^−6^) in this study. The hybrid initialization technique is used for numerical simulation.

The SARS coronavirus-2 aerosol deposition condition at the airway wall is used as a “trap” (Ghosh *et al.*, 2020, Islam *et al.*, 2019), which means, during inhalation, if the particle touches the airway wall, the system will count it as deposited. The velocity of the SARS coronavirus-2 aerosol particles will be zero at the airway wall as the wall is considered static, and the aerosol trajectory will terminate at the wall. The deposition efficiency (DE) of the SARS coronavirus-2 aerosol is based on the ratio of the trapped aerosol and total injected aerosol.

## III. GEOMETRICAL DEVELOPMENT

**FIG. 1.**
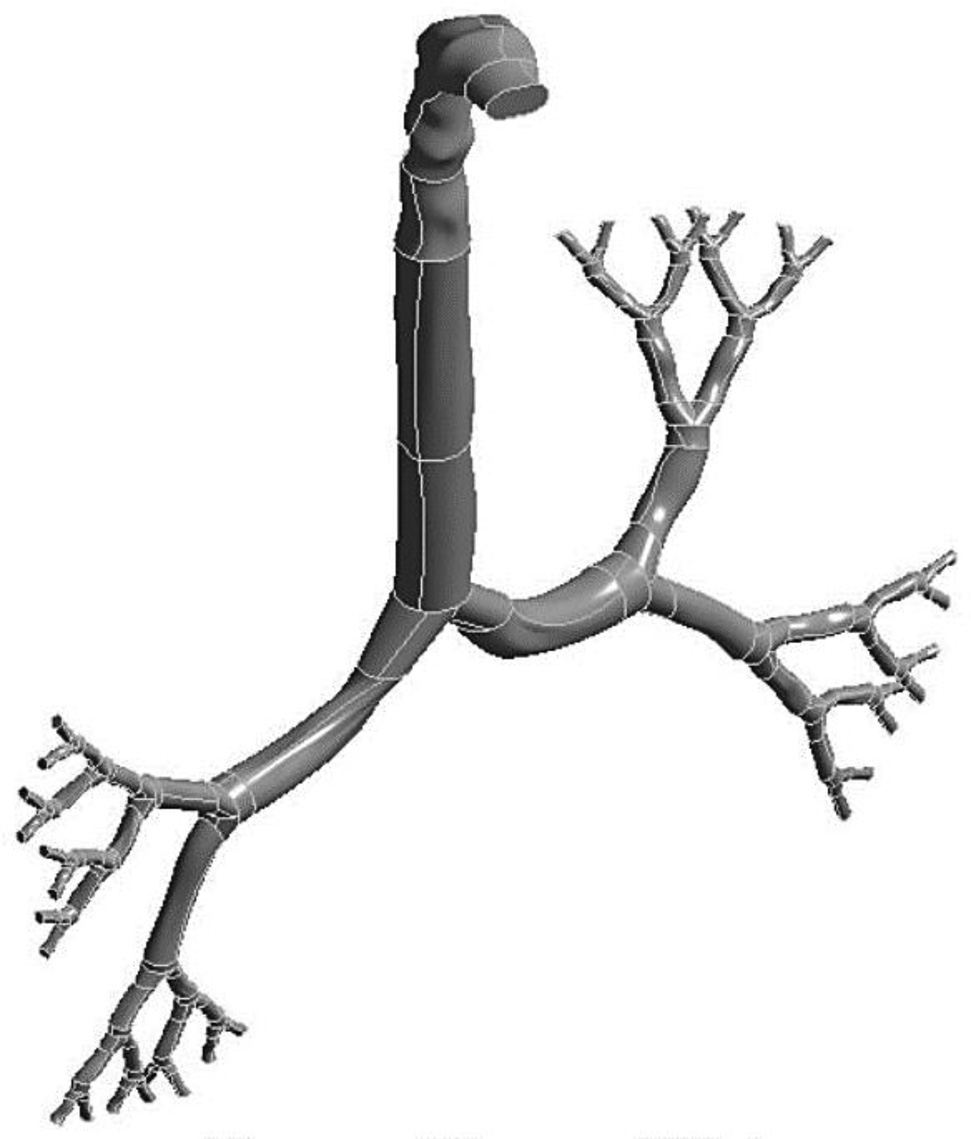
Reconstruction airway model for the lung of a 50-year-old subject.

In the present study, three anatomical models were constructed for 50-, 60-, and 70-year-old people. The anatomical models in this study contain computed tomography (CT)-scan based mouth–throat and Weibel's based (Zhu *et al.*, 1963) reconstructed tracheobronchial airway (from the trachea to the first–fifth generations). AMIRA and Geomagic softwares are used to visualize the CT-Dicom images, and three-dimensional anatomical models are developed. For the tracheobronchial airways, SolidWorks software is used. An earlier study analyzed the morphology of the human lung due to aging and showed 10% reduction in the airway size (Niewoehner and Kleinerman, 1974). Later, an analytical equation was developed by Xu and Yu (1986) for the age-specific lung upon the findings of Niewoehner and Kleinerman (1974). Recently, a computational study analyzed the airflow in an age-specific lung and reduced the airway dimension by 10% for people over 50 years (Kim *et al.* 2017). The airway dimension, shape, and branching pattern could vary from person to person, depending on age. This study used the data available in the literature along with a 10% reduction for people over 50 years. However, in reality, the airway dimension could be different for various ages people depending on age, sex, and other physiological conditions. Figure 1 shows the reconstructed airway model for the 50-year-old lung. For the 60- and 70-year-old models, airway dimensions are reduced by 10%.

## IV. MESH GENERATION, MESH REFINEMENT, AND VALIDATION

**FIG. 2.**
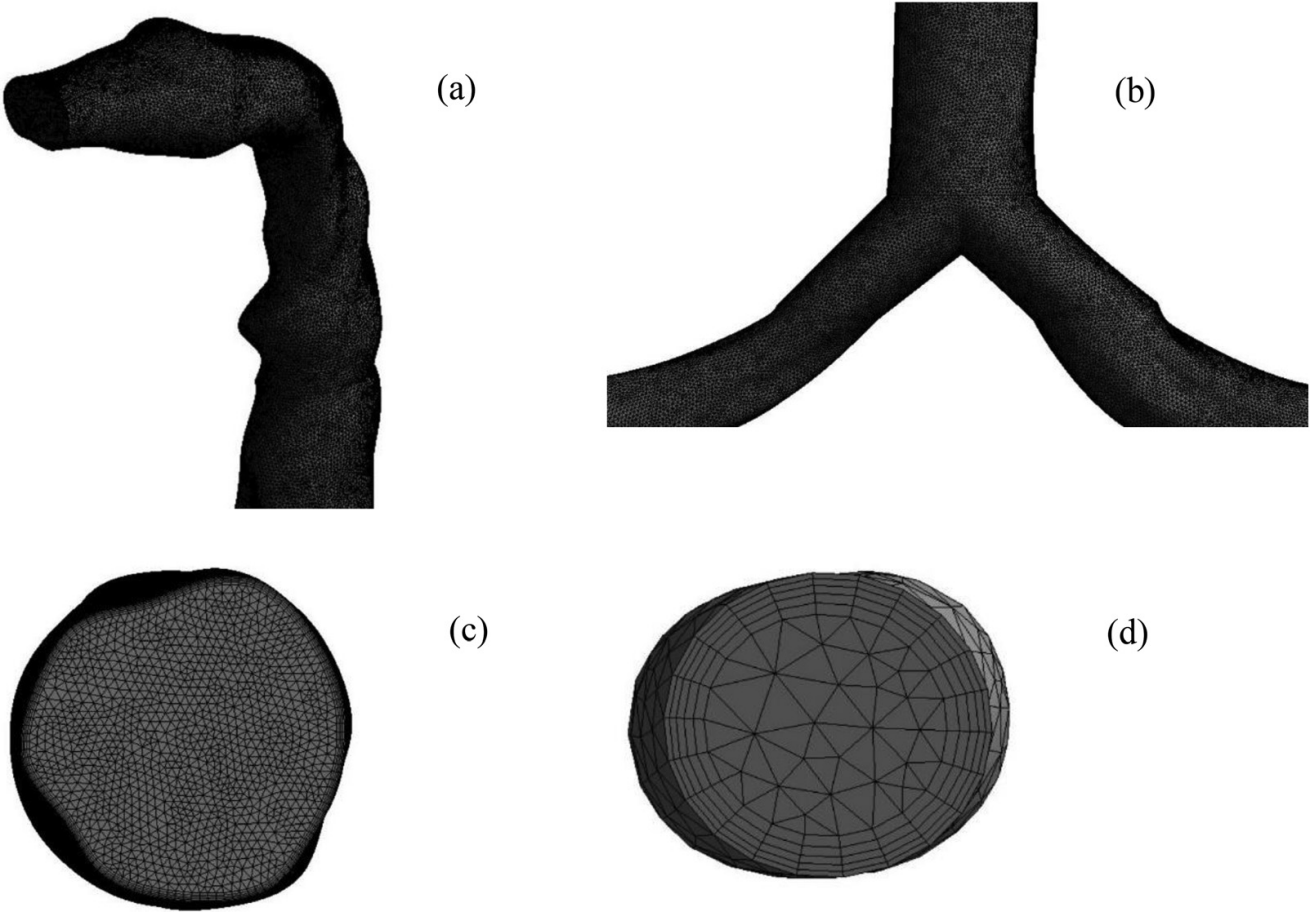
Computational mesh for the airway; (a) mouth–throat section, (b) first bifurcation, (c)inflation mesh at mouth area, and (d) inflation mesh at the outlet.

**FIG. 3.**
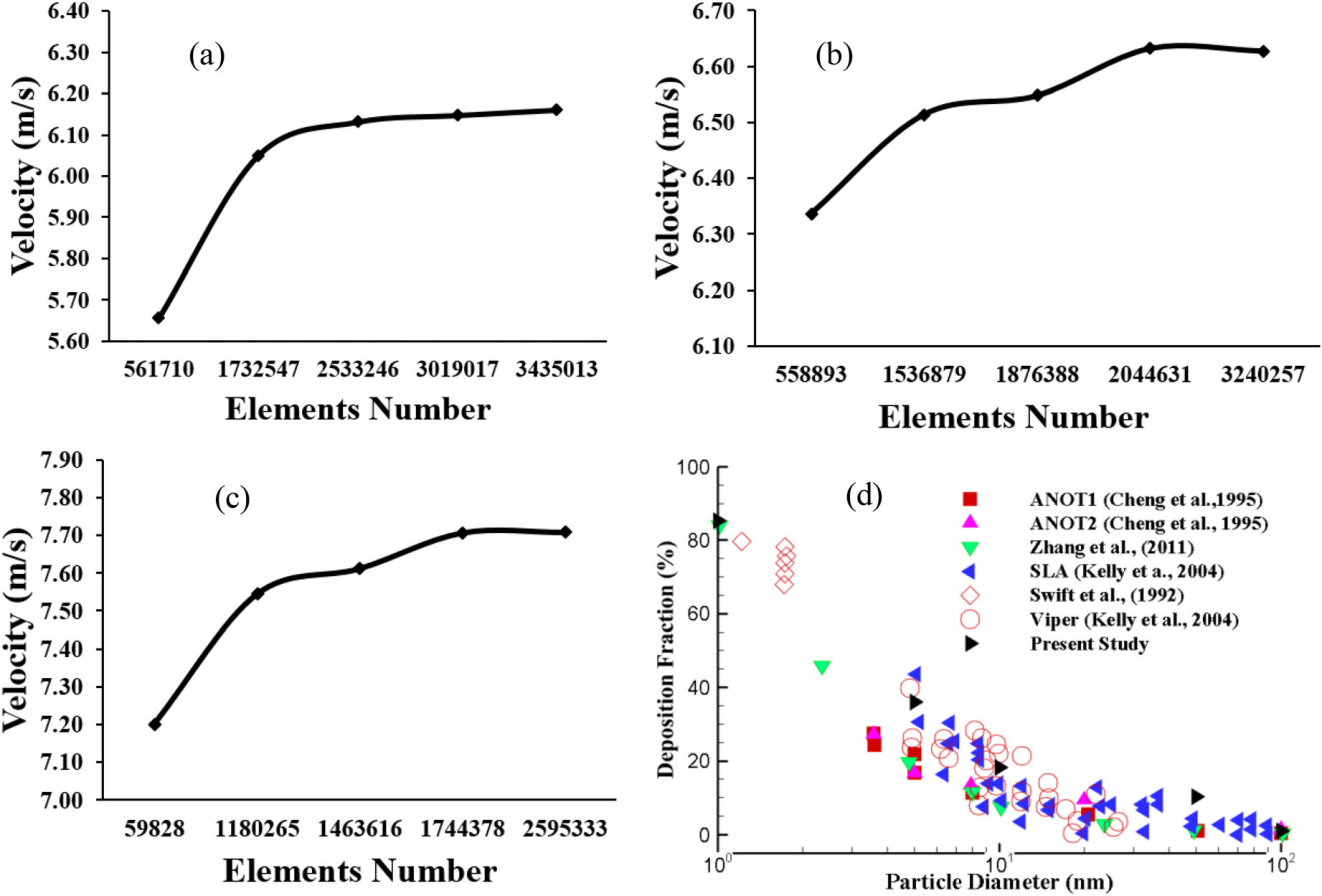
Grid refinement results for (a) 50-, (b) 60-, (c) 70-year-old, and (d) deposition fraction comparison with the available literature at a flow rate of 10 lpm (Cheng *et al.*, 1995a; Cheng and Swift, 1995b; Kelly *et al.*, 2004; Swift *et al.*, 1992; Zhang and Kleinstreuer, 2011).

The Ansys meshing module is employed for the computational mesh, and unstructured tetrahedral mesh elements are generated for the three different lung models. The inflation layer is generated adjacent to the airway wall, and the bifurcation area consists of highly dense mesh elements (Srivastav *et al.*, 2019b). Figure 2(a) presents the mesh at the mouth region, and Fig. 2(b) presents the mesh element at the first bifurcation area for the 50-year-old model. Figures 2(c) and 2(d) show the inflation layer mesh at the mouth and outlet region of the lung model. A detailed grid refinement is performed, and Fig. 3 shows the grid convergence results. The final computational model for the 50-year-old lung model consists of 2.5 × 10^6^ cells, while the 60- and 70-year-old models consist of 2 × 10^6^ and 1.7 × 10^6^ cells, respectively. The aged model provides the steady solution for the lower number of cells as the airway diameter is reduced.

The CFD model is authenticated with the available experimental and computational measurements. The deposition fraction at the upper airway is compared with the available literature findings (Cheng *et al.*, 1995a; Cheng *et al.*, 1995b; Kelly *et al.*, 2004; Swift *et al.*, 1992; Zhang and Kleinstreuer, 2011). The present study used a 50-year-old model for the deposition fraction comparison. The present computational results closely align with the published measurement, which eventually supports the present computational study results.

## V. RESULT AND DISCUSSION

**FIG. 4.**
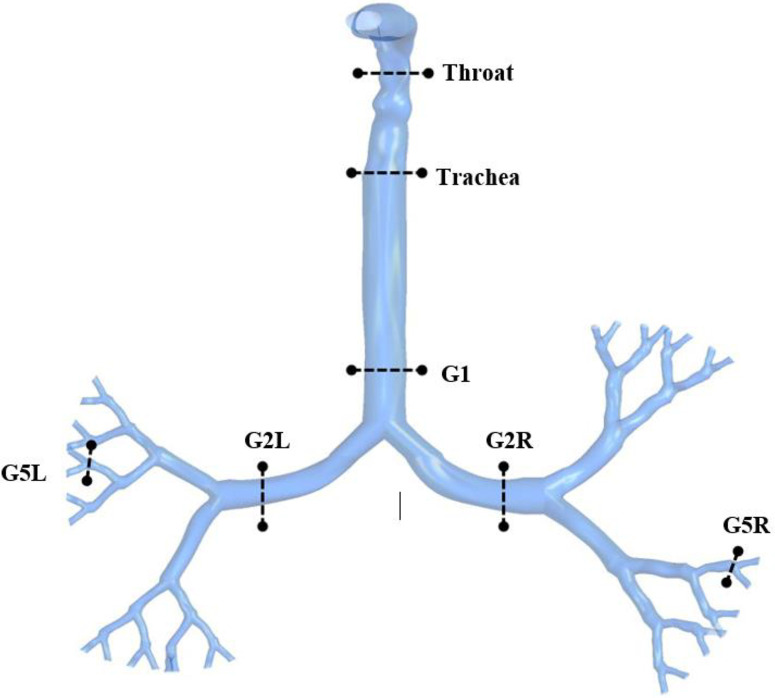
Selected planes for velocity contours at various branches.

**FIG. 5.**
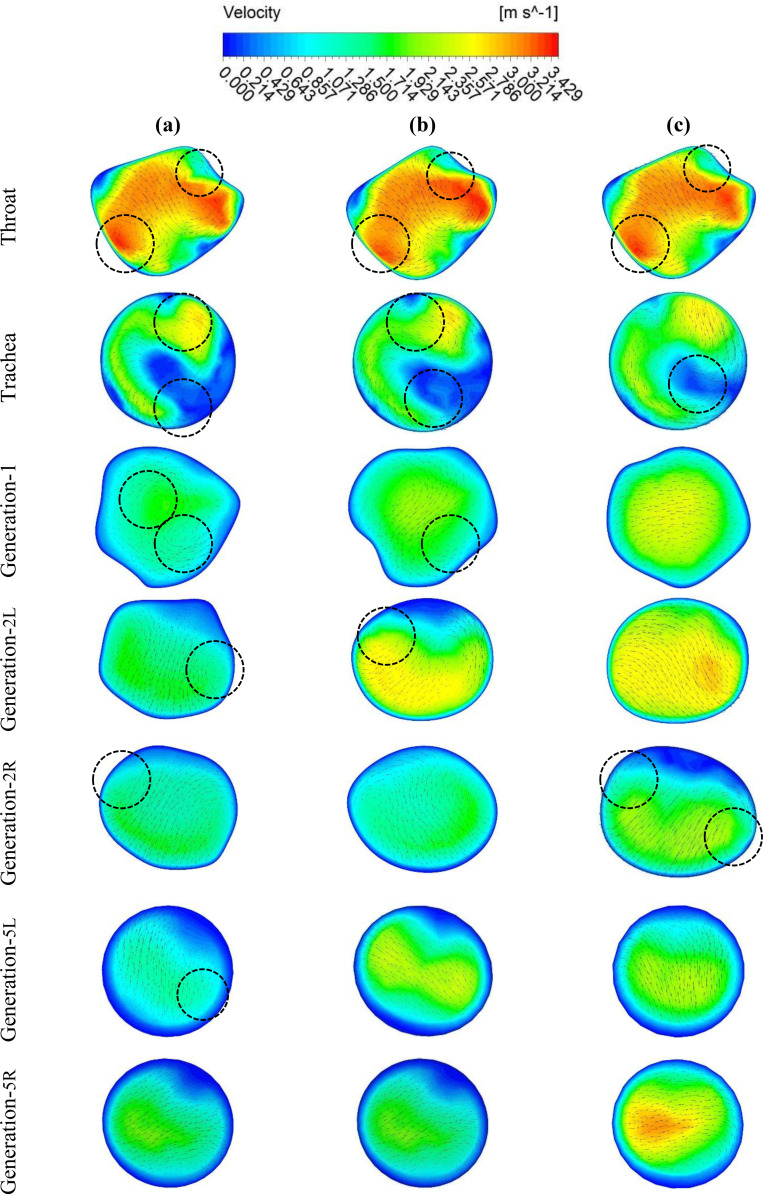
Velocity contours at various positions of the pulmonary models at 15 lpm for **(a)** 50-years old subject, **(b)** 60-years old subject, and **(c)** 70-years old subject.

Figure 5 presents the velocity contours at selected locations (see Fig. 4) of the upper airways from the mouth–throat area to the fifth generation. These contours represent the velocity fields and velocity vectors at the 15 lpm inlet condition. Figures 5(a)–5(c) show the contours for 50-, 60-, and 70-year-olds, respectively. For 50-year-olds, more complex flows with two vortices are found at the throat, trachea, and generation 1. However, the rest contours show that only one vortex has been formed for both sides of pulmonary generation 2 and the left side of generation 1. Moreover, it can be observed from the figure that no vortex is generated for the right lung in generation 5. For 60-year-olds, the throat and tracheal areas have more complex flows with two vortices. There is only one vortex solution at generation 1 and the left lung at generation 2. For 70-year-olds, the throat and the right lung at generation 2 generate more complex flows with two vortices following by the one vortex solution at the trachea. In terms of the velocity magnitude, the highest velocity is located at the throat area for all three cases. For 50-year-olds, the velocity continuously decreases along with the airway generations. For 60-year-olds, the velocity becomes higher at the left lung of generation 2 and decreases along with the generations. For 70-year-olds, the velocity decreases from the throat area to generation 1 and then increases at the left lung of generation 2 and the right lung of generation 5. The overall velocity and vector contours indicate that the 50-year-old case consists of different flow behaviors for most generations compared to the other two cases. However, the 70-year-old case has higher velocity magnitudes from generation 1 to lower generation than 1. In terms of velocity patterns, only the throat and trachea have similar flow fields in all downstream generations.

**FIG. 6.**
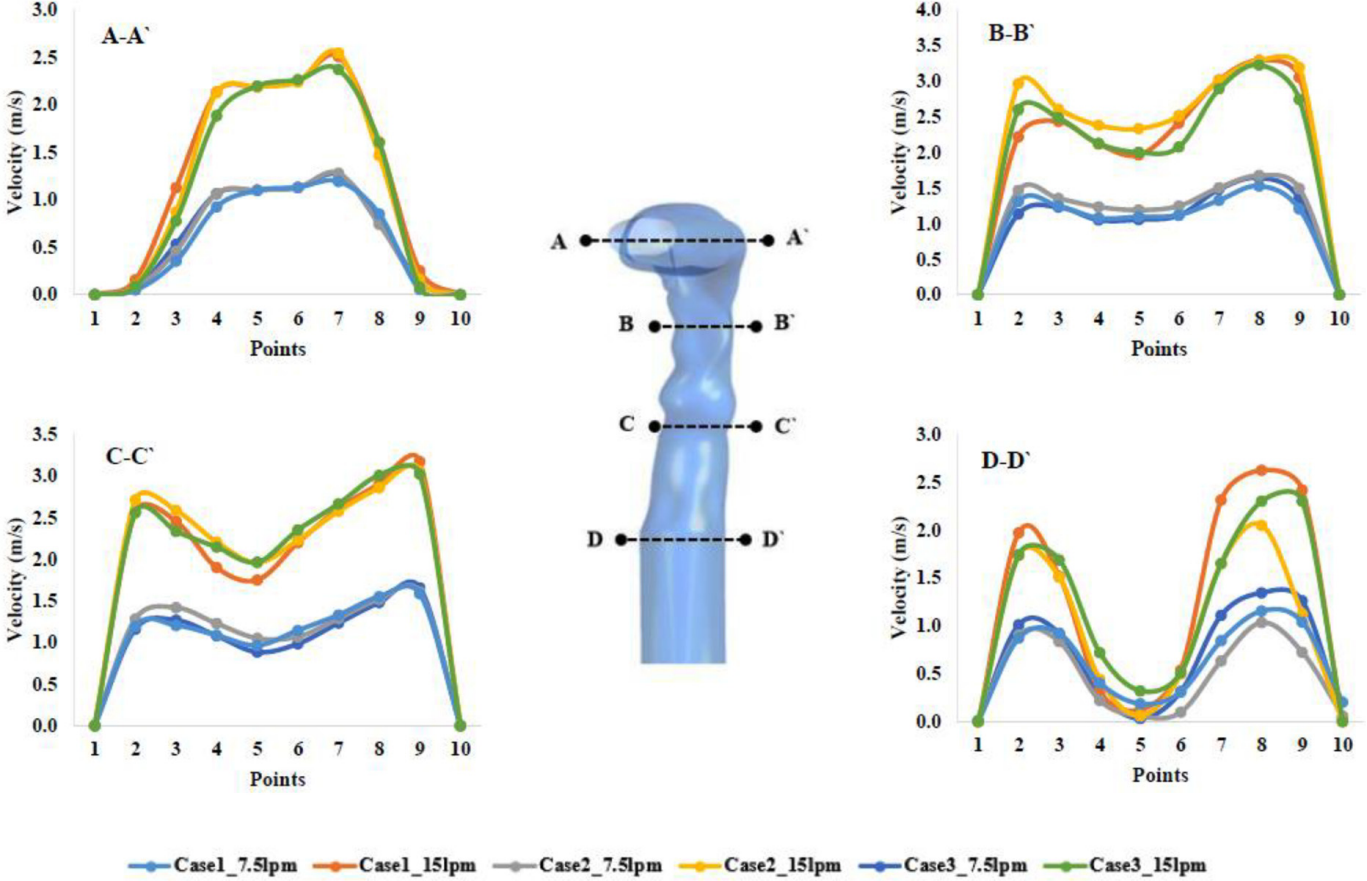
Velocity profiles at various positions of mouth area for three lung models with different flow rates. Case 1: 50-years; case 2: 60-years; case 3: 70-years old subject.

Figures 6(a)–6(d) represent the velocity profile across a straight line at different cross sections along the throat to the trachea. According to Figs. 6(a)–6(d), the velocity profiles for different cases at lines A, B, C, and D show that the velocity magnitude increase with the flow rate in the central area of the related lines. However, the flow profile across lines A, B, C, and D is not identical. Figures 6(a)–6(d) show that the velocity magnitude in the proximity to the centerline of the airway decreases from the throat to the trachea. This behavior of velocity magnitude stems from the presence of the secondary flow, which is generated due to the bending airways at the throat. Figure 6(d) shows that the velocity in the center of the trachea is less than 0.5** **m/s, which is much lower than other noncentral areas. Hence, it can be implied that the airflow in the upper airways passes the out-of-central area of the airway dominantly. Therefore, it is anticipated that SARS coronavirus aerosol deposition on the wall of the trachea decreases since the velocity is high in the out-of-central area.

**FIG. 7.**
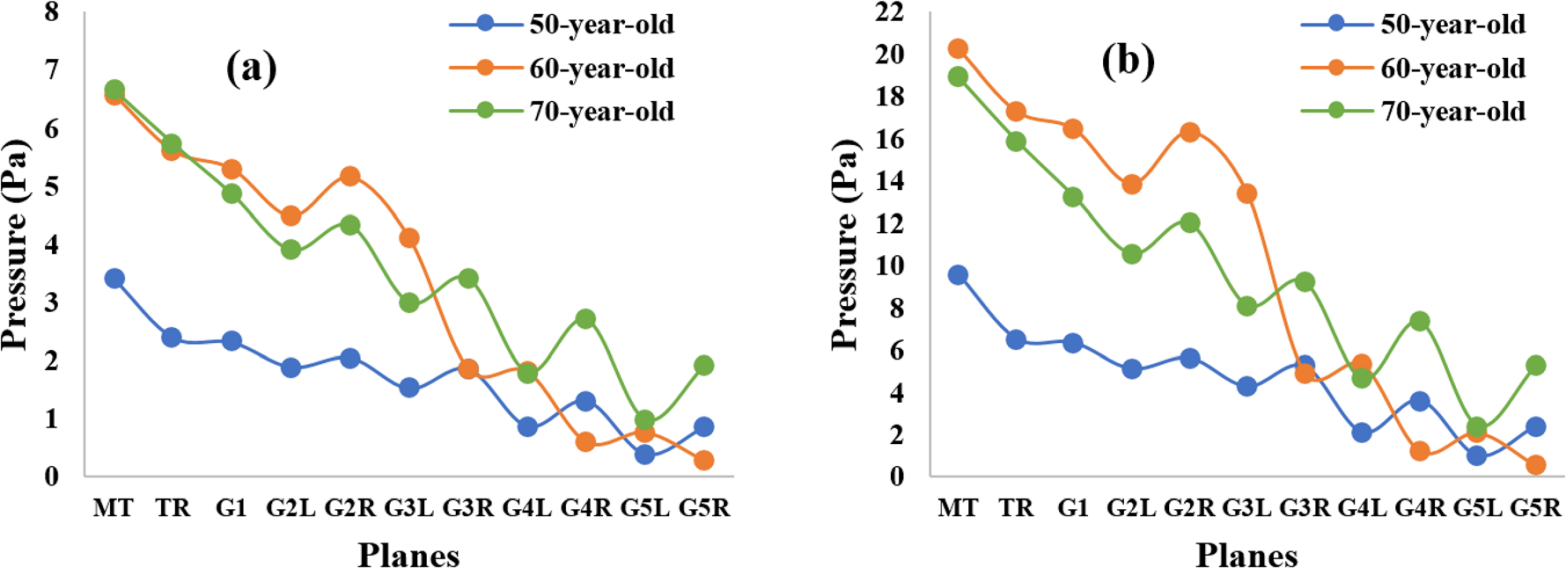
Pressure at various selected positions for three lung cases at (a) 7.5 lpm and (b) 15 lpm. MT, mouth–throat; TR, trachea; G1, generation 1; G2L, generation 2-left lung; G2R, generation 2-right lung; G3L, generation 3-left lung; G3R, generation 3-right lung; G4L, generation 4-left lung; G4R, generation 4-right lung; G5L, generation 5-left lung; G5R, generation 5-right lung.

Figures 7(a) and 7(b) show the pressure distribution along the airways, starting from throat to generation 5 for all age-specific cases under differing inlet conditions of 7.5 and 15 lpm. It is obvious from this figure that the pressure value decreases in the lower airways compared to the upper airways. This pressure drop generates the airflow in human airways. According to Fig. 7, the pressure value for 50-year-olds is lower than for 70** **year-olds in all locations in airways. The pressure value for 60-year-old subjects is higher than other subjects in areas from mouth–throat to generation 3. The pressure value in generation 5 of 60-year-old subjects is lower than that for other-aged subjects. When the inhalation rate is 7.5 lpm, the pressure drop between the mouth and generation 5 for 60-year-old subjects is about 6.5** **Pa, while the pressure drops for the 50- and 70-year-old subjects are about 2 and 3.5** **Pa, respectively. Overall, it implies that the pressure drops as the human lung ages.

**FIG. 8.**
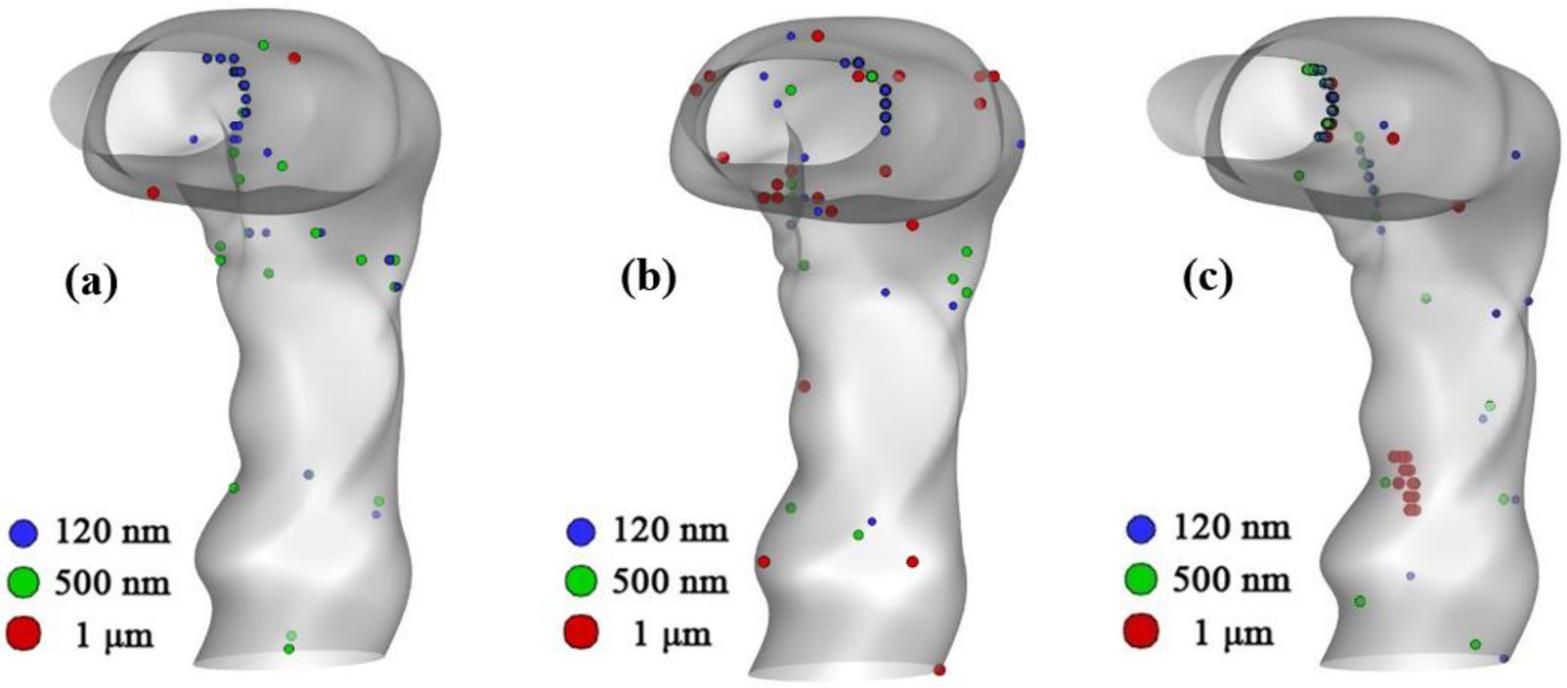
Deposition scenario in the mouth–throat area at 15 lpm (a) for a 50-year-old subject, (b) for a 60-year-old subject, and (c) for a 70-year-old subject.

**FIG. 9.**
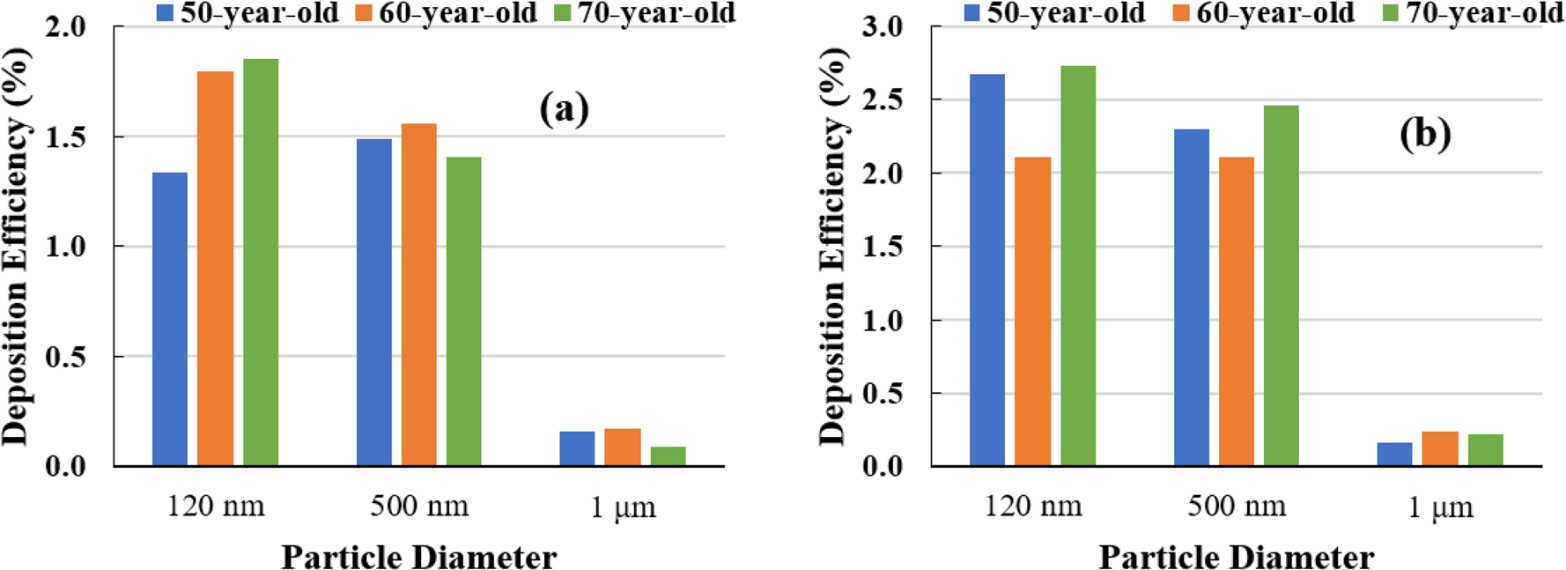
Particle DE comparison in the mouth–throat area for three lung models (a) at 7.5 lpm and (b) at 15 lpm.

The deposition of the SARS coronavirus-2 aerosol in the human upper and lower airways is representative of the transport behavior of COVID-19 within the human airways. Figure 8 represents the deposition of different sized SARS coronavirus-2 aerosols at the mouth–throat area. Figures 9(a) and 9(b) show the efficiency of SARS coronavirus-2 aerosol deposition in the mouth area for various sizes at inhalation rates of 7.5 and 15 lpm, respectively. According to Figs. 9(a) and 9(b), in the mouth area, smaller SARS coronavirus-2 aerosol deposition is greater than larger SARS coronavirus-2 particles for all models. Figure 9(a) shows that at the 7.5 lpm case, the DE of smaller SARS coronavirus-2 aerosol (120 nm) in the mouth–throat area increases with aging; however, for a bigger SARS coronavirus-2 aerosol (i.e., 500 nm), the DE of 60-year-old subjects is higher than for the other two 50- and 70-year-old subjects. This relationship between the DE and the age of the subject occurs for micro-sized SARS coronavirus-2 aerosols as well. The Brownian motion is the main mechanism for smaller diameter aerosols. The low inlet velocity, smaller diameter aerosol, and highly asymmetric airway model increase the deposition concentration at the mouth–throat section. In contrast, at 15 lpm, the DE of the SARS coronavirus-2 aerosol in the mouth–throat area for 60-year-old subjects is lower than for 50- and 70-year-olds. Comparing Figs. 9(a) and 9(b), the DE of larger SARS coronavirus-2 aerosols for 60-year-old subjects is greater than 50- and 70- year-old subjects under both flow conditions. However, by increasing the age from 50 to 70, the DE of the larger SARS coronavirus-2 aerosol (1 *μ*m) decreases when the inhalation rate is 7.5 lpm, while it increases at the inhalation rate of 15 lpm.

**FIG. 10.**
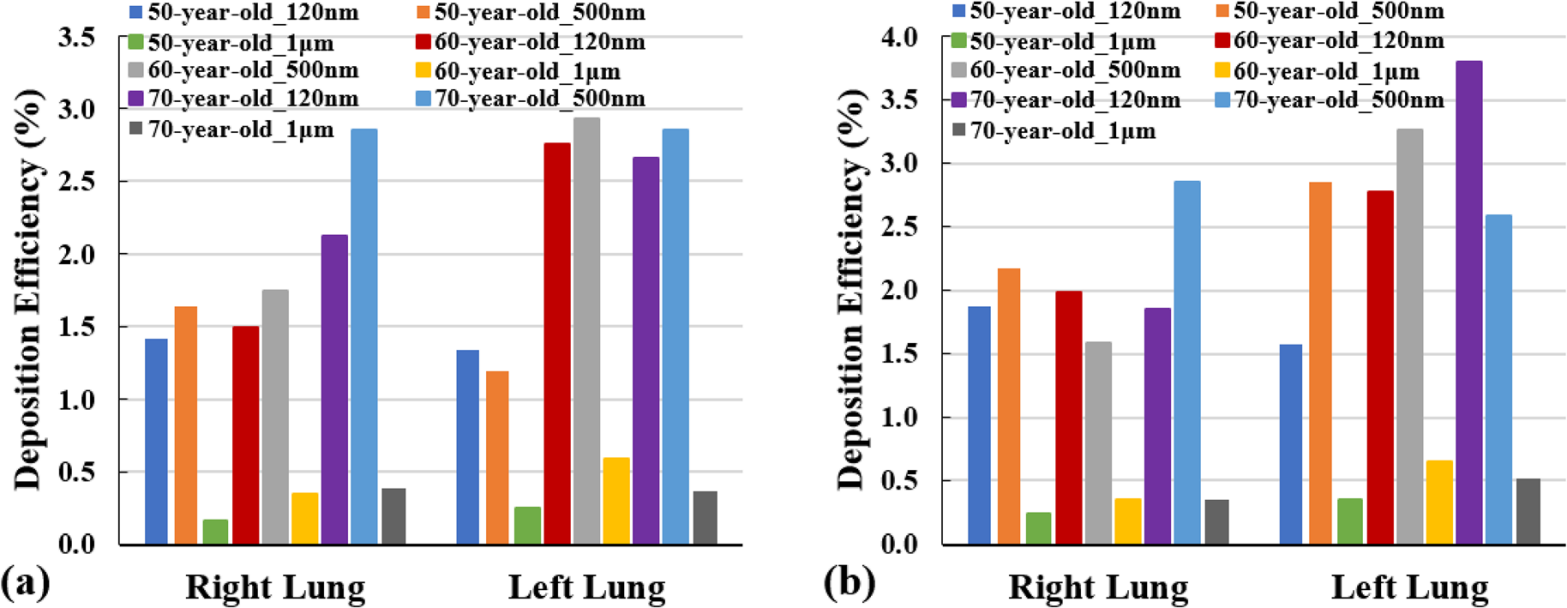
SARS coronavirus-2 aerosol DE at right and left bronchioles (a) for 7.5 lpm and (b) for 15 lpm.

Figure 10 illustrates the SARS coronavirus-2 aerosol DE in the left and right lungs at different inlet flow rates. As can be seen from Fig. 10(a), the lowest DE for all cases occurs when SARS coronavirus-2 aerosols are in the microscale (i.e., 1 *μ*m). When the flow rate is 7.5 lpm, the highest DE in the right lung corresponds to the 60-year-old subject with the aerosol diameter of 500 nm. Also, the DE in the right lung for 500 nm SARS coronavirus-2 increases with the increasing age of the subjects. Figure 10(a) also shows that when the inhalation rate is 7.5 lpm, the DE decreases considerably with the increasing aerosol size for the youngest subjects (i.e., 50-year-olds). Figure 10(b) demonstrates that the DE in the right lung decreases significantly with the increasing particle size for 60-year-old subjects when the inhalation rate is 15 lpm. This behavior between the aerosol particle size and DE can also be seen in the left lung for an older subject (70-year-old). According to Fig. 10, the highest DE in the left lung occurs for the oldest subject, i.e., 70-year-old under the inlet condition of 15 lpm with is about 3.5%. Figure 10 also demonstrates that the DE of the larger SARS coronavirus-2 aerosol (1 *μ*m) in the right lung has an increasing trend with aging under both inhalation conditions.

**FIG. 11.**
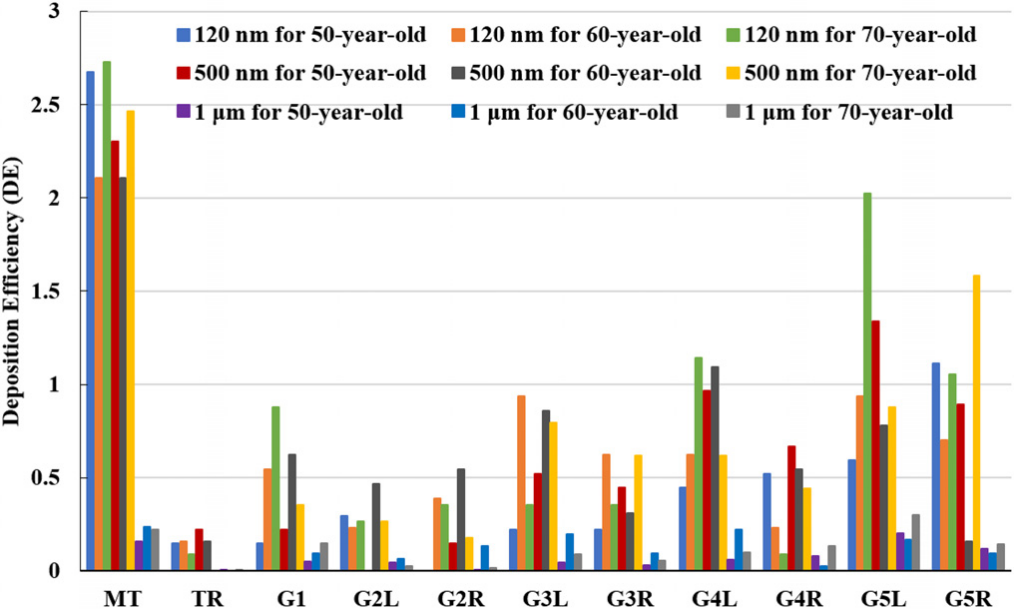
Deposition hot spot comparison for different diameters at 15 lpm of inlet conditions.

Figure 11 presents the DE at different locations from the mouth–throat region to the fifth generation of human airways for different particle sized SARS coronavirus-2 aerosols and for various age-specific subjects for flows of 15 lpm. It is clear from this figure that the highest DE occurs in the mouth–throat region, while the DE lowers in the tracheal region. The high deposition in the mouth–throat regions is due to this area's bending shape, which exerts a centrifugal force on the particles passing through the throat. The lower DE in the trachea occurs due to its cylindrical shape where no sudden change in the flow direction occurs. On the other hand, the DE increases along the airways from the trachea to the tracheobronchial airways of the lung. Among the generations, the downstream generation in this study, i.e., generation 5, has the highest DE, which is due to the fact that when generations progress toward the lung, the diameter of the airways decreases, and the majority of the particles comes closer to the walls and the possibility of particle deposition increases. Figure 11 also shows that a decrease in the particle diameter increases the local DE in generations 1, four-left, and five-left. Moreover, this figure demonstrates that the lowest generation of the left lung (i.e., generation 5) has the highest DE for the oldest subject with an aerosol diameter of 120 nm.

**FIG. 12.**
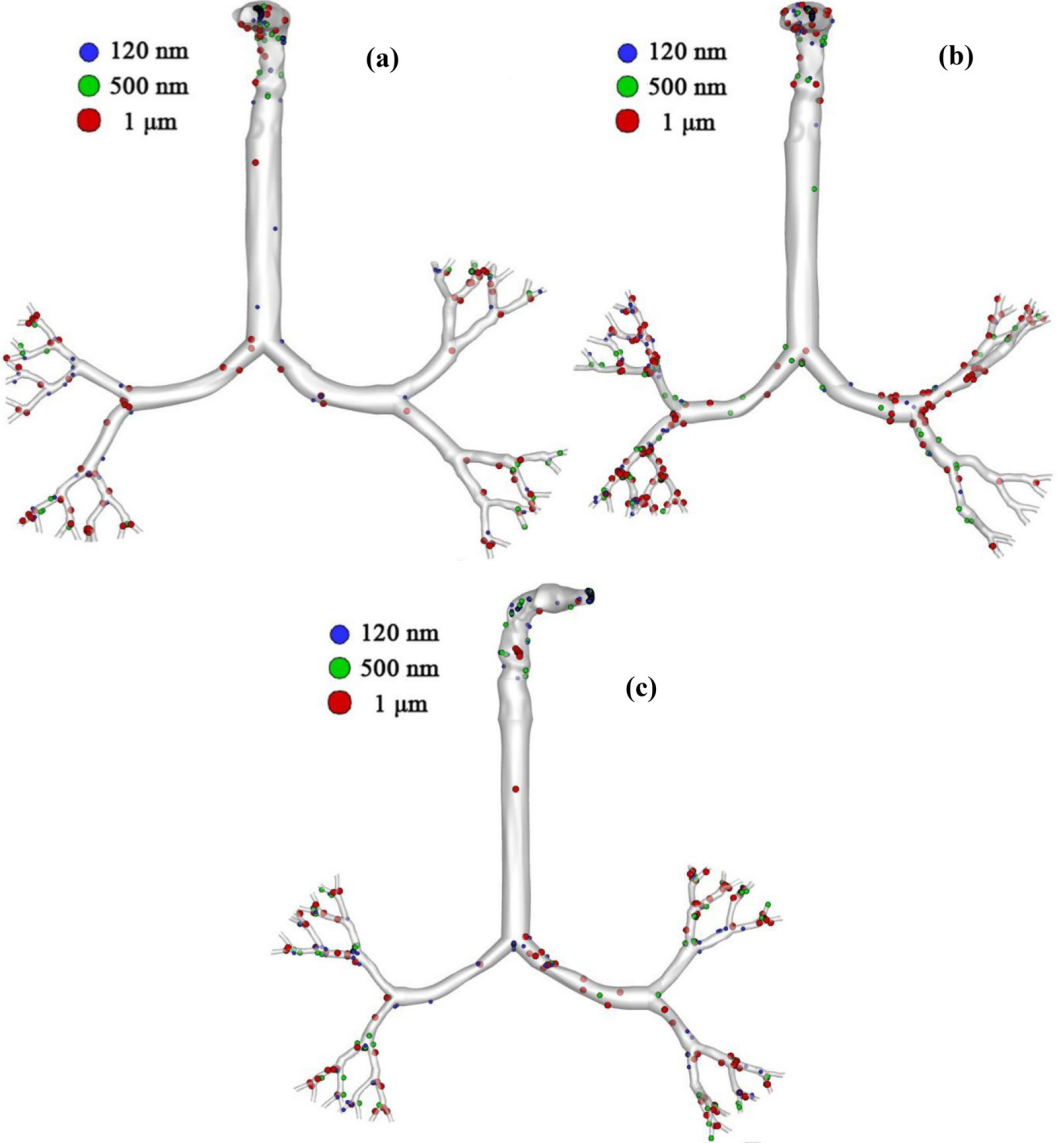
Particle deposition scenario at the flow rate of 15 lpm for (a) 50-year-old, (b) 60-year-old, and (c) 70-year-old.

Figure 12 shows an overview of particle deposition in the whole geometry from mouth to the fifth generation. From this figure, it can be seen that SARS coronavirus-2 aerosols are mostly deposited at the mouth–throat and bifurcation area of the upper airway models. The overall deposition scenario shows higher SARS coronavirus-2 aerosol depositions for aged subjects than for the younger ones. The general deposition pattern shows smaller diameter SARS coronavirus-2 aerosol particles are mostly deposited in the upper airways than are the larger sized SARS coronavirus-2 aerosol particles. It is evident from the literature that Brownian motion is the principal mechanism of smaller diameter (nano) particle transport in the human bronchial tree (Farhadi Ghalati *et al.*, 2012; Ghosh *et al.*, 2020; Zhang and Kleinstreuer, 2004). Brownian motion will be more dominant when the inhalation rate is minimum and the random movement of the smaller diameter aerosol particles increases the deposition concentration in the upper airways (Islam *et al.*, 2017b). Under low inlet conditions, smaller diameter aerosols randomly move inside the airways, and the spontaneous movement increases the deposition at the highly asymmetric mouth–throat and upper airways.

## VI. CONCLUSIONS

SARS coronavirus-2 aerosol depositions are numerically investigated for the first time for age-specific lungs. Three upper lung airway models for 50-, 60-, and 70-year-olds are developed, based on various SARS coronavirus-2 aerosol particle sizes and flow rates. An inclusive analysis is performed for the airflow and pressure variations throughout the upper airways (generations 1 to 5). A comprehensive generation-by-generation SARS coronavirus-2 aerosol DE is calculated and presented. Some key findings of the study are summarized below as follows:

• The general pressure variation from mouth–throat to the fifth generation of the upper airway model increases with age. In the 7.5 lpm case, the highest pressure is observed for 70-year-old lungs, while the lowest pressure is observed for 50-year-old lungs. On the contrary, the 15 lpm inhalation case results indicate the highest pulmonary pressure occurs for the 60-year-old model.
• The SARS coronavirus-2 aerosol DE for smaller diameter SARS coronavirus-2 aerosols is higher than that of the larger diameter SARS coronavirus-2 aerosols, irrespective of the inhalation conditions.
• The overall DE of the SARS coronavirus-2 aerosols for aged lungs is found higher than for the younger counterparts. Almost all cases (irrespective of flow conditions and aerosol particle sizes) show higher depositions for 70-year-old lungs than for 50-year and 60-year-old counterparts.
• SARS coronavirus-2 aerosols demonstrate a highly complex deposition pattern in the right and the left lungs, respectively. At the 7.5 lpm flow rate, smaller diameter SARS coronavirus-2 aerosol particle deposition in the right lung is greater than in the left lung. On the contrary, larger diameter SARS coronavirus-2 aerosol particle deposition is higher in the left lung than in the left lung. A similar trend is observed for 15 lpm inhalation conditions for various particle diameters of SARS coronavirus-2 aerosols.
• The SARS coronavirus-2 aerosol shows high concentration at the mouth–throat region for all deposition parameters than in the bifurcating branches. The aerosol deposition concentration is also found to be higher at the fifth generation of the left and the right lungs.
• The overall DE of the SARS coronavirus-2 aerosol increases from upper generations to lower generations (i.e., from generation 1 to 5).

In this study, SARS coronavirus-2 deposition at the mouth–throat and upper airways of the age-specific lung was critically analyzed and led to the identification of a high-concentration deposition zone. In the study, a range of SARS coronavirus-2 aerosol diameter was considered. The specific findings of this study would improve the knowledge of SARS coronavirus-2 aerosol transport to the upper airways of the age-specific lung. The comprehensive generation-by-generation deposition data for an age-specific lung could be useful for the health risk assessment of the SARS coronavirus-2 affected aged people.

No conflicts of interest are reported.

## Data Availability

The data that support the findings of this study are available from the corresponding author upon reasonable request.
